# Bortezomib modulates CHIT1 and YKL40 in monocyte-derived osteoclast and in myeloma cells

**DOI:** 10.3389/fphar.2015.00226

**Published:** 2015-10-14

**Authors:** Daniele Tibullo, Michelino Di Rosa, Cesarina Giallongo, Piera La Cava, Nunziatina L. Parrinello, Alessandra Romano, Concetta Conticello, Maria V. Brundo, Salvatore Saccone, Lucia Malaguarnera, Francesco Di Raimondo

**Affiliations:** ^1^Section of Hematology, Department of Surgery and Medical Specialties, University of Catania, Catania, Italy; ^2^Department of Biomedical and Biotechnology Sciences, University of Catania, Catania, Italy; ^3^Department of Biological, Geological and Environmental Sciences, University of Catania, Catania, Italy

**Keywords:** osteoclasts, chitinases, multiple myeloma, U266, SKM-M1, CHIT1, YKL40, bortezomib

## Abstract

Osteolytic bone disease is a common manifestation of multiple myeloma (MM) that leads to progressive skeleton destruction and is the most severe cause of morbidity in MM patients. It results from increased osteolytic activity and decrease osteoblastic function. Activation of mammalian chitinases chitotriosidase (CHIT1) and YKL40 is associated with osteoclast (OCs) differentiation and bone digestion. In the current study, we investigated the effect of two Bortezomib’s concentration (2.5 and 5 nM) on osteoclastogenesis by analyzing regulation of chitinase expression. OCs exposition to bortezomib (BO) was able to inhibit the expression of different OCs markers such as RANK, CTSK, TRAP, and MMP9. In addition BO-treatment reduced CHIT1 enzymatic activity and both CHIT1 and YKL40 mRNA expression levels and cytoplasmatic and secreted protein. Moreover, immunofluorescence evaluation of mature OCs showed that BO was able to translocate YKL40 into the nucleus, while CHIT1 remained into the cytoplasm. Since MM cell lines such as U266, SKM-M1 and MM1 showed high levels of CHIT1 activity, we analyzed bone resorption ability of U266 using dentin disk assay resorption pits. Silencing chitinase proteins in U266 cell line with specific small interfering RNA, resulted in pits number reduction on dentine disks. In conclusion, we showed that BO decreases osteoclastogenesis and reduces bone resorption in OCs and U266 cell line by modulating the chitinases CHIT1 and YKL40. These results indicate that chitinases may be a therapeutic target for bone disease in MM patients.

## Introduction

Multiple myeloma (MM) is a clonal B-cell malignancy characterized by accumulation of clonal plasma cells (PCs) in the bone marrow (BM) leading to bone destruction and BM failure. Osteolytic bone disease is a common manifestation of MM that leads to a progressive destruction of the skeleton. This is the most severe cause of morbidity because of pathological fractures, spinal cord compression, chronic bone pain and extreme disability (Mundy, 1998; Terpos et al., 2013). Pathogenetic mechanisms of MM bone destruction are closely linked to MM PC and osteoclasts (OCs) hyperactivity coupled with defective osteoblast (OB) function unable to counteract bone resorption. It has been demonstrated that malignant PC alter the cellular composition of the bone (Noll et al., 2014). MM cells promote osteoclastogenic effect both by exerting themselves bone destruction and through recruitment, differentiation and activation of OC progenitors within the BM (Calvani et al., 2005). In response to PC, tumor associated stromal cells, that physiologically differentiate into OBs produce numerous pro-osteoclastogenic factors increasing OC recruitment and OC-mediated bone loss at sites proximal to the PC tumor (Farrugia et al., 2003; Heider et al., 2005; Zannettino et al., 2005). OCs are large multinucleated cells that arise from precursors of the mononuclear-phagocytic lineage through increased levels of receptor activator of nuclear factor κB ligand (RANKL) and M-CSF, whose intracellular pathways propagate signals that activate sequential transcription factors, resulting in the production of major OC enzymes that drive specific functions such as acidification and degradation of the bone matrix (Fujikawa et al., 1996; Bahar et al., 2007; Silvestris et al., 2009). Many studies have demonstrated an increase of RANKL, the major osteoclast-activating factors, in the MM BM microenvironment (Sezer et al., 2002; Heider et al., 2003). In OCs, RANKL/RANK binding activates TRAF6 (tumor necrosis factor receptor-associated factor 6) which activates AP-1 (activator protein-1), NF-κB (nuclear factor kappa B), and p38 MAPK inducing the transcription of OCs promoting genes (von Metzler et al., 2007). The regulation of the transcriptional activity of AP-1 and NF-κB transcription factors also occurs by the proteasome-ubiquitin system. A constitutively increased proteasome activity has been reported in myeloma cells (Jakob et al., 2007). Bortezomib (BO) is the first proteasome inhibitor approved for treatment of MM patients. The drug induces the stabilization of NF-κB antagonist I-κB and AP-1 transcription factors c-Fos and c-Jun resulting in reduction of OCs differentiation (von Metzler et al., 2007; Hongming and Jian, 2009).

Mammalian chitinases belong to the glycohydrolase family 18, which have evolved to hydrolyze chitin, a polymer of *N*-acetylglucosamine (Herrera-Estrella and Chet, 1999). The family of chitinases includes members both with and without glycohydrolase enzymatic activity against chitin. Chitotriosidase (CHIT1) and acidic mammalian chitinase (CHIA or AMCAse) are the only two true chitinase possessing chitinolytic (glycohydrolase) activities (Boot et al., 2001). In contrast, chitinase-like-lectins (Chi-lectins) or chitinase-like proteins (C/CLPs), including chitinase 3-like-1 (CHI3L1, YKL40, HC-gp39), chitinase 3-like-2 (CHI3L2, CHIL2, YKL-39), chitinase domain containing 1 (CHID1), show enzymatic activity despite the retention and conservation of the substrate-binding cleft of the chitinases (van Aalten et al., 2001). Elevated levels of chitinases have been reported in a variety of diseases including infections, chronic inflammation and degenerative disorders (Malaguarnera, 2006; Di Rosa and Malaguarnera, 2012). In particular, a recent study showed that the activation of alternative macrophage is involved in osteolysis and suggested a correlation between CHIT1 and osteolytic lesions (Koulouvaris et al., 2008). Moreover, it was observed that in patients with myeloma elevated serum concentrations of YKL40 worsened bone destruction and were associated with an increase of bone resorption activity hastening the progression of bone disease (Mylin et al., 2008). In rat model of cold I/R injury, some authors demonstrated that the upregulation of YKL-40 is a mechanisms responsible for steatotic liver I/R injury and this can be abrogated with the administration of BO (Tiriveedhi et al., 2014).

A recent study of our group demonstrated that CHIT1 and YKL40 have a crucial role in osteoclastogenesis and in osteolysis mediated by matrix metalloproteinase-9 (MMP9; Di Rosa et al., 2014). In the current work, we investigated the effect of BO on chitinases expression during osteoclastogenesis. In addition, we also evaluated the expression of chitinases in MM cell lines founding a their direct role in plasma cells-mediated bone resorption *in vitro*.

## Materials and Methods

### Cells

The U266, SKM-M1, and MM1 were obtained from American Type Culture Collection (Manassas, VA, USA). The cell lines were maintained in RPMI medium containing 2 mM l-glutamine, supplemented with 10% fetal bovine serum (FBS) and intermittently with 100 U/ml penicillin and 100 μg/ml streptomycin at a humidified 37°C incubator providing 5% CO_2_. Human monocytes were isolated, after informed consent, from fresh buffy coat of healthy volunteers provided by the Transfusional Centre “Garibaldi” Hospital, Catania, S. Immuno-Haematology and Transfusional Medicine. Monocytes then were purified from the lymphomonocytic population by positive isolation using magnetic beads coated with goat anti-mouse CD14+ IgG (Miltenyi Biotec GmbH, Germany). Analysis of monocytes was performed by multicolor FACS (Cytomics FC 500, Beckman Coulter) using the following antibodies (Beckman Coulter): anti-CD14 and anti-CD11c. Monocytes, identified as CD14+ CD11c+ cells, showed purity greater than 90% (data not showed).

### *In Vitro* OCs Differentiation

Monocytes isolated from PBMCs were cultured at a density of 5 × 10^5^ cells/cm^2^ in 24-well culture plates in conditioned IMDM supplemented with 10% FBS, 2 mM glutamine, and 1% of penicillin/streptomycin (Invitrogen, Milan, Italy). In order to obtain OCs, the conditioned medium was supplemented with 25 ng/ml soluble rhRANK ligand (PeproTech. BDA, Italy) and 20 ng/ml rhM-CSF (PeproTech. BDA, Italy), for 21 days w/o BO (2.5 or 5 nM). The medium was replaced every 3 days and supernatants were harvested for enzymatic assay. To confirm that macrophages achieved OCs differentiation, suitable markers were analyzed by real-time PCR (qRT-PCR). Finally, in order to evaluate the ability of MM cell lines (U266) to digest bone, dentine disks were added to the wells before cell seeding. U266 cultured with conditioned medium (without BO) for 24 h were used as a control.

### Gene Expression Analysis by Real-Time PCR

After RNA extraction and reverse transcription, we evaluated expression of the following mRNA: CHIT1 (Hs00185753_m1), YKL40 (Hs01072228_m1), MMP9 (Hs00234579_m1), CTSK (Hs00166156_m1), TRAP (Hs00356261_m1). Their expression was assessed by TaqMan Gene Expression (Applied Biosystem) and quantified using a fluorescence-based real-time detection method by 7900HT Fast Start (Applied Biosystem). For each sample, the relative expression level of each studied mRNA was normalized using GAPDH (Hs02758991_g1) as invariant controls.

### Western Blot

Cells were harvested by trypsinization and total proteins were extracted using M-PER cell lysis buffer (THERMO Scientific, USA). The lysates were collected for Western blot analysis. Protein concentrations were determined according to the Bradford method.

Protein levels were visualized by immunoblotting with antibodies against human CHIT1 (sc-99033, Santa Cruz Biotechnology, USA), YKL40 (sc-30465, Santa Cruz Biotechnology, USA), MMP9 (sc-13520, Santa Cruz Biotechnology, USA) and GAPDH (sc-365062, Santa Cruz Biotechnology, USA). Briefly, 30 μg of lysate supernatant was resolved by SDS/polyacrylamide gel electrophoresis on 4–20% Mini-PROTEAN^®^ TGX^™^ (BIO-RAD, Milan, Italy) and transferred to a nitrocellulose membrane *trans*-Blot Turbo mini nitrocellulose (BIO-RAD, Milan, Italy) using a semidry transfer apparatus (BIO-RAD, Hercules, CA, USA). The membranes were incubated with 5% milk in 10 mM Tris-HCl (pH 7.4), 150 mM NaCl, 0.05% Tween 20 (TBST) buffer at 4°C overnight. After washing with TBST, the membranes were incubated with a 1:2000 dilution of anti-CHIT1, anti-YKL40, anti-MMP9 or anti-GAPDH antibodies for 1 h at room temperature with constant shaking. The filters were then washed and probed with horseradish peroxidase-conjugated anti-rabbit IgG-HRP (Santa Cruz Biotechnology, USA) for CHIT1, donkey anti-goat IgG-HRP (Santa Cruz Biotechnology, USA) for YKL40 and MMP9 and goat anti-mouse IgM-HRP (Santa Cruz Biotechnology, USA) for GAPDH at a dilution of 1:2000. Chemiluminescence detection was performed with the Amersham ECL detection kit according to the manufacturer’s instructions.

### Determination of Supernatant Proteins

Culture supernatants (100 μl) were collected and stored at –20°C, at the indicated time points and used for protein extraction. Briefly 900 μl of 100% isopropanol was added and centrifuged at 12,000 × *g* for 15 min. The pellet was dissolved in M-PER (100 μl) and solubilized proteins (5 μl, ∼20 μg) from all samples were electrophoresed on 12% SDS/polyacrylamide gel and transferred to nitrocellulose membrane to perform Western blot analysis.

### Immunofluorescence

Cells were grown directly on coverslips before immunofluore-scence. After washing with phosphate-buffered saline (PBS), cells were fixed in methanol–acetone (1:1) for 10 min at –20°C and then washed three times for 5 min in PBS. Subsequently, coverslips were incubated with the primary antibody anti-CHIT1 (Santa Cruz Biotechnology, USA) and anti-YKL40 (Santa Cruz Biotechnology, USA) diluted 1:400 in PBS containing 0.1% Tween 20 and 1% BSA overnight at +4°C. Cells were washed as above before incubation with secondary antibody, Alexa Fluor 594-conjugated Affini Pure goat-anti-rabbit IgG for CHIT1 and Alexa Fluor 594-conjugated Affini Pure donkey-anti-goat IgG for YKL40, diluted 1:500 in PBS and 0.1% Tween 20 and 1% BSA, then washed once more as above. Finally, coverslips were mounted on microscope slides with Ultracruz TM anti-fade medium containing 4′,6-diamidino-2-phenylindole (DAPI; Santa Cruz Biotechnology). Localization of CHIT1 and YKL40 was then performed by using a confocal laser scanning microscopy (CLSM; Zeiss LSM700).

### Chitotriosidase Activity Assay

Chitotriosidase enzymatic activity was determined in cell-free supernatants of the OCs at different stages of differentiation. The activity was determined by a fluorimetric method using 22 μM 4-methylumbelliferyl β D-NNN-triacetylchitotriosidase (Sigma Chemical Co.) in citrate–phosphate buffer, pH 5.2, as previously described (Di Rosa et al., 2005).

Fluorescence was read at 450 nm with a Perkin Elmer LS40 fluorimeter (excitation wavelength 365 nm). As control, cell-free supernatants of monocytes cultured for 24 h were used. Enzymatic activity was measured as nanomoles of substrate hydrolyzed per ml per hour (nmol/ml/h).

### Cell Transfection and siRNA

Multiple myeloma cell lines (U266) were cultured in six-well plates at a density between 2.5 and 5 × 10^5^ cells/ml for 24 h. Cells were transfected with small interfering RNA (siRNA; 20 nM) targeting CHIT1 (Santa Cruz Biotechnology, Inc.) and YKL40 (Santa Cruz Biotechnology, Inc.) in the presence of siRNA transfection reagent (Santa Cruz Biotechnology, Inc.), according to the manufacturer’s instructions. Control cells were transfected with scrambled random siRNA (Santa Cruz Biotechnology, Inc.). The cells were incubated for 96 h before collection.

### Resorption Assay

The U266 cell line and the OCs cells (5 × 10^4^/cm^2^) were plated onto dentine disks (Osteosite Dentine Disks, Immunodiagnostic Systems Inc., Fountain Hills, AR, USA) in 96-well plates for the digestion test. After 24 h, the cells were treated with siRNA, as described above in materials and methods. After 96 h, the cells were removed with 5% sodium hypochlorite for 10 min. Disks were rinsed with water and stained with 1% (w/v) toluidine blue in 0.5% sodium borate for 30 s and then washed twice with water. Individual pits or multiple pit clusters were observed and counted using a microscope at 25–100 × magnification. Four microscopic field observation were used to count the number of resorption pits. Results were expressed as the number of resorption pits. Experiments were performed in triplicate.

### The 3-(4,5-Dimethylthiazolyl-2)-2,5-Diphenyl-Tetrazolium Bromide (MTT)

The MTT assay was performed to detect cell viability. Briefly, cells were seeded in 96-well plates at 1 × 10^3^ cells/well, and then were treated with siRNAs as previously described. After 48 h of siRNA treatment, 20 μl of MTT solution (5 mg/ml) was added to each well. After 3 h of incubation, formazan crystals were dissolved in 150 μl of 0.1 M HCl in isopropanol. Color intensity was measured at 570 nm with ELISA plate reader (THERMO Multiskan FC).

### Statistical Analysis

Data are expressed as mean ± standard error (SD). Significance was assessed by ANOVA or Student’s *t* test. *p*<0.05 was considered to be statistically significant.

## Results

### BO Inhibits OCs Differentiation

**FIGURE 1:**
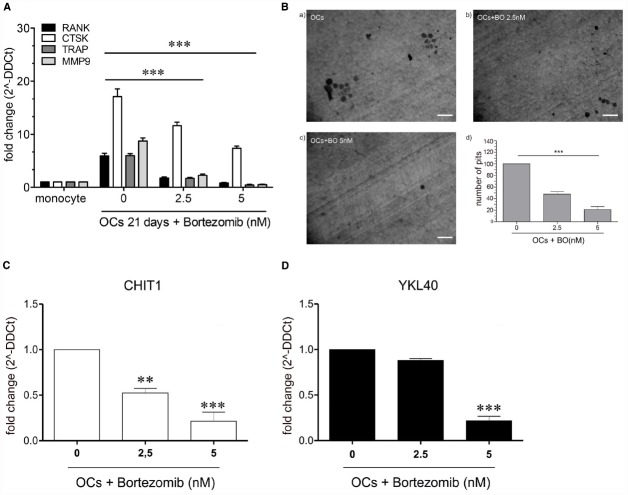
**The figure shows a significant reduction (***p***<0.0001) of OCs marker in the cells treated with 2.5 and 5 nM with BO compared to the negative control (OCs not treated) (A).** Determination of resorption pits on dentin disk showed a significant reduction of bone matrix digestion (47.65%, *p*=0.005) in the cells treated with BO 2.5 nM and in the cells treated with BO 5 nM (21%, *p*=0.005), compared to the control (OCs without BO) **(B)**. The treatment with BO determined a modulation of chitinases. CHIT1 mRNA expression was decreased in a dose-dependent manner in presence of BO (2.5 nM) respect to monocytes without BO (ctrl; *p*<0.003) and BO 5 nM (*p*<0.003). BO was capable of decrease also the mRNA expression of YKL40, about onefold at BO 2.5 nM and with a significantly decrease with BO 5 nM (about fivefold, *p*<0.003) **(C,D)**. Data are expressed as mean ± SD of at least three independent experiments. **P<0.01, ***P<0.001.

To detect whether BO was able to modulate the process of OC differentiation, we treated monocytes with RANKL (25 ng/ml), MCSF (20 ng/ml) and w/o BO for 21 days. Cell viability did not change after BO exposure (data not shown). Expression of OCs markers (MMP9, RANK, CTSK, and TRAP) and bone matrix digestion were investigated after 21 days of culture. We detected a significant reduction both of OCs markers expression (*p*<0.0001) and of the ability of bone digestion in OCs after BO exposure (Figures 1A,B).

We recently demonstrated that CHIT1 and YKL40 have a crucial role in osteoclastogenesis. To this aim, we explored the effect of BO on CHIT1 and YKL40 expression in monocytes after 21 days from the beginning of OC differentiation. We found that BO led to a down-regulation of both the mRNA in a dose-dependent manner (*p*<0.001 and *p*<0.0001 respectively at 2.5 and 5 nM of BO for CHIT1; *p*<0.0001 at 5 nM for YKL40; Figures 1C,D), suggesting that BO inhibits osteoclastogenesis through, at least in part, downregulation of chitinases expression.

### BO Affects Both Cytoplasmic and Secretory Component of CHIT1 and YKL40 Protein

**FIGURE 2:**
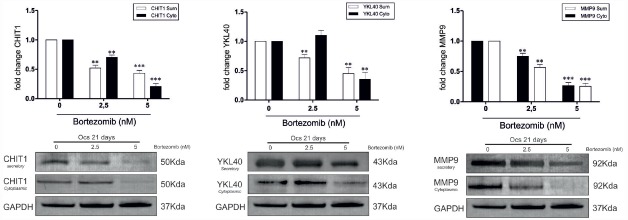
**In OCs treated with BO the chitinases protein expression was significantly decrease for the two isoforms of CHIT1 (39 kDa lysosomal and 50 kDa secretory), for the two component of YKL40 (50 kDa cytoplasmatic and secretory) and for the two component of MMP9 (92 kDa cytoplasmatic and secretory) when BO was added to culture.** All results are compared to OCs without BO. Values are shown as means ± SD of three independent experiments. The figure shows representative data from one of three replicate experiments **P<0.001, ***P<0.0001.

Since it has been reported that MMP9 expression is closely associated with CHIT1 and YKL40 production (Malaguarnera et al., 2009; Di Rosa et al., 2014), we next investigated what component (cytoplasmic and secretory) was affected by BO treatment after 21 days from the beginning of OC differentiation. We found that BO was able to induce a significant decrease of both the components of CHIT1, YKL40, and MMP9 in a dose-dependent manner, with the exception of the cytoplasmic component of YKL40 at a dose of 2.5 nM (Figure 2).

### BO Modulates CHIT1 Activity During OCs Differentiation

**FIGURE 3:**
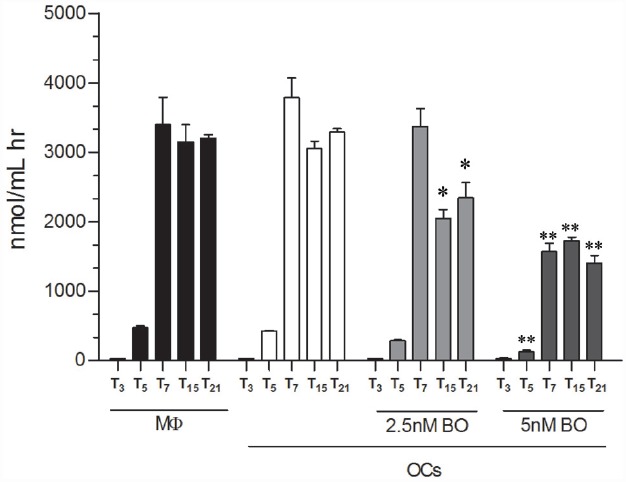
**CHIT1 enzyme activity was measured in cell-free supernatants.** Culture supernatants were analyzed, enzyme activity calculated and expressed as nanomoles per liter per hour. Data are expressed as mean ± SD of at least three independent experiments. *P<0.01, **P<0.001 compared to the respective time in OCs without BO.

Afterward, we evaluated whether the secreted CHIT1 was enzymatically active in OCs after 3, 5, 7, 15, and 21 days from the beginning of differentiation and BO exposure. We found that 2.5 nM BO significantly decreased CHIT1 activity of 34 ± 8 and 29 ± 8% at 15 and 21 days (*p*<0.01; Figure 3). Five nM BO was able to decrease the protein activity during all differentiation process (70 ± 5, 62 ± 1, 65 ± 4, and 68 ± 4% respectively at 5, 7, 15, and 21 days; *p*<0.001).

### Subcellular Distribution of CHIT1 and YKL40 in OCs After Exposure to BO

**FIGURE 4:**
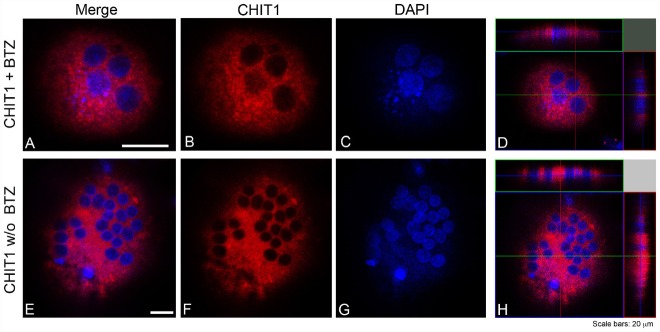
**CHIT1 was detected with Alexa Fluor 594-conjugated Affini Pure Goat-anti-Rabbit IgG (Red).** Most of the cells in immunostaining for CHIT1 show a characteristic distribution pattern, the signal being localized only in the cytoplasm. The Blue represents DAPI nuclear counterstain. It is important to note the many nuclei present per cell. Scale bars equal 20 μm. **(A,E)** Double staining of CHIT1 in red and nuclei in blue. **(B,F)** CHIT1 staining in red alone. **(C,G)** nuclear staining with DAPI alone. **(D,H)** Z-stack confocal images, double staining of CHIT1 in red and nuclei in blue.

**FIGURE 5:**
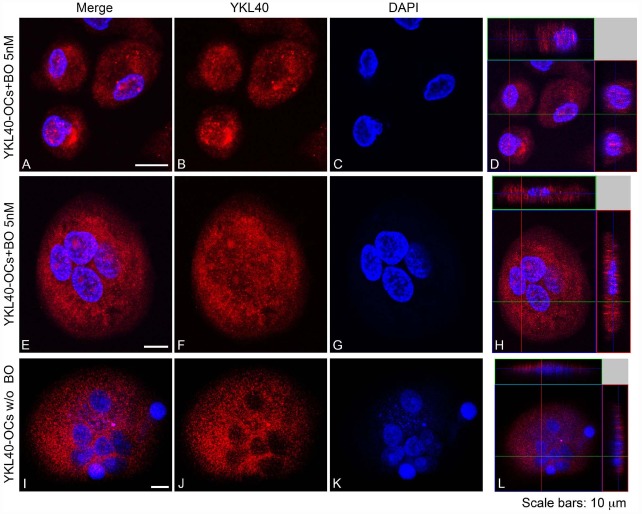
**YKL40 was detected with Alexa Fluor 594-conjugated Affini Pure Goat-anti-Rabbit IgG (Red).** Most of the cells in immunostaining for YKL40 show a characteristic distribution pattern, the signal being localized in the cytoplasm and in the nucleo in cell treated with BO. The Blue represents DAPI nuclear counterstain. It is important to note the many nuclei present per cell. Scale bars equal 10 μm. **(A,E,I)** Double staining of YKL40 in red and nuclei in blue. **(B,F,J)** CHIT1 staining in red alone. **(C,G,K)** nuclear staining with DAPI alone. **(D,H,L)** Z-stack confocal images, Double staining of YKL40 in red and nuclei in blue.

Our data suggest that BO inhibits osteoclastogenesis through, at least in part, downregulation of YKL40 and CHIT1 expression and activity. We further looked at the subcellular distribution of CHIT1 and YKL40 by immunofluorescence in OCs after 21 days from the beginning of differentiation to examine if their localization could undergo a change after BO exposure. Because the maximum modulation of chitinases was observed with 5 nM BO, we chose this dose as a point of reference for the assay. Immunofluorescence analysis showed the characteristic morphology of OCs that are multinucleated large size-cells (about 200 μm; Figure 4D). CHIT1 and YKL40 distribution were purely cytoplasmic (Figures 4H and 5L). Treatment with BO led to a reduction of cells size and nuclei numbers (Figures 4A and 5E). No nuclear localization was detected for CHIT1 after BO exposure (Figure 4). On the contrary, BO treatment was able to induce nuclear translocation of YKL40 (Figures 5D,H). Remarkably, the protein localized into the nucleus after BO exposure also in mononuclear cells (Figure 5D). Thus, these data indicated that BO was also able to affect YKL40 car localization.

### BO Modulates CHIT1 and YKL40 Expression and Activity in MM Cell Lines

**FIGURE 6:**
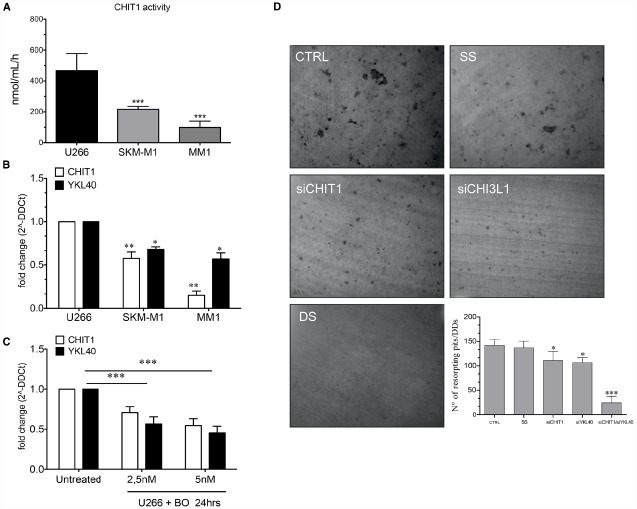
**The MM cells line (U266, SKM-M1, and MM1) express different levels of CHIT1 activity (A) and chitinase mRNA expression CHIT1 and YKL40 (B).** The treatment of U266 with BO (2.5 and 5 nM) for 24 h reduce the mRNA expression of CHIT1 and YKL40 **(C)**. Determination of resorption pits on dentin disk showed a significant reduction in the cells treated with CHIT1 siRNA, YKL40 siRNA and in the co-silencing with CHIT1 and YKL40 siRNA (DS) compared to the negative control (SS) **(D)**. Results are expressed as the number of resorption pits. Data are expressed as mean ± SD of at least three independent experiments.*P<0.01, **P<0.001, ***P<0.0001.

Since recent observations suggest that malignant plasma cells take part in bone destruction (Silvestris et al., 2009), we next analyzed CHIT1 activity in MM cell lines (U266, SKM-M1, and MM1). High activity levels were found with a mean value of 466 ± 111.29, 216 ± 19.07, and 98 ± 41.44 respectively for U266, SKM-M1, and MM1 (Figure 6A). Since U266 showed the highest CHIT1 activity levels, we analyzed CHIT1 and YKL40 mRNA expression in MM cell lines using U266 as control. Also chitinases expression resulted higher in U266 than SKM-M1 and MM1 cells (Figure 6B); therefore, we chose U266 as cellular model to evaluate effect of BO on CHIT1 and YKL40 expression in MM cells. We found that BO was able to down-regulate CHIT1 and YKL40 mRNA expression in a dose-dependent manner (*p*<0.0001; Figure 6C), suggesting that BO may impair PC ability to participate in bone resorption.

Next, we evaluated the capacity of bone matrix digestion by U266 and the involvement of CHIT1 and YKL40 in the digestive process. The cells were plated onto dentin disks (DDs) and divided into different groups: U266 alone (control); cells transfected with scrambled random siRNA (SS; negative control); cells transfected with CHIT1 or YKL40 siRNAs and U266 co-transfected with both siRNAs. Digestive activity was observed both in DDs with U266 alone and in the negative control group (Figure 6D). Treatment with CHIT1 and YKL40 siRNAs alone or in combination, resulted in a significant reduction of about 21.98 ± 5.2 and 25.08 ± 3% in the digestion activity compared to the negative control (*p*<0.003). Interestingly, inhibition of both CHIT1 and YKL40 led to a decrease of 82.85 ± 7% in the digestion activity of about compared to negative control (*p*<0.0001). Taken together these data strongly suggest that PC act as functional OCs through expression and activity of chitinases.

## Discussion

It has been known that OCs and their activators are very important for myeloma cell growth and survival (Hongming and Jian, 2009). Terpos et al. (2006) reported that BO treatment significantly reduced serum DKK-1, sRANKL as well as bone resorption markers including TRAP-5b and CTX, promoting normalization of bone remodeling in MM patients.

Recent studies have shown that BO treatment determines a reduction of osteoclastogenesis and bone digestion in MM patients (von Metzler et al., 2007; Hongming and Jian, 2009). This inhibitory effect is likely linked to the ability of the drug to block the proteasome activity and activate NF-κB pathways (Qiang et al., 2012). We here demonstrated, for the first time, that BO inhibits osteoclastogenesis also through downregulation of CHIT1 and YKL40 in a dose-dependent manner, leading to reduction of bone resorption. In fact, chitinases not only are crucial during the osteoclastogenesis process, but are also directly involved in the digestive process (Di Rosa et al., 2014).

First, we confirmed that BO exposure during OC differentiation led to the inhibition of OC markers expression and reduced the bone digestion ability of OCs in a dose-dependent manner. We further showed that drug treatment down-regulated expression of CHIT1 and YKL40 in a dose-dependent manner. In particular, BO affected both the component (cytoplasmic and secretory) of CHIT1, YKL40 and MMP9, whose expression is closely associated with chitinases production. Also CHIT1 activity was affected by BO during OCs differentiation. This reduction is closely related with down-regulation of mRNA and secretory/lysosome CHIT1 protein levels.

Treatment with BO during OC differentiation modulated also the expression of YKL40. This chitinase does not have the catalytic site but it is able to bind chitin through its chitin binding domain (CBM). It has been demonstrated that the expression of YKL40 is linked to the NF-κB pathways (Recklies et al., 2005; Bhat et al., 2008; Tang et al., 2013) and this could explain the observed reduction of YKL40 in OCs treated with BO. Many reports have shown that high levels of plasma YKL40 are associated with increased bone lesions in patients with MM (Mylin et al., 2006, 2008, 2009), but its role is not yet understood. Recently, our group has demonstrated that silencing YKL40 with siRNA in human mature OCs results in decreased levels of the matrix degrading collagenase MMP9 as well as decreased bone resorptive activity *in vitro*, suggesting a key role for YKL40 in OC function (Di Rosa et al., 2014). In accordance with our findings, Mylin et al. (2015) demonstrated that serum YKL-40 is a new independent prognostic marker for skeletal complications in MM patients.

We also observed and reported for the first time that YKL40 is able to translocate into the nucleus in OCs exposed to BO during differentiation. We can hypothesize that a structural change of the cytoskeleton induced by BO leads YKL40 to bind F-actin (Poglazov and Livanova, 1986).

Silvestris et al. (2009) has recently discussed the role of malignant PCs in bone disease of MM patients, demonstrating that PCs produced pits on experimental osteologic substrates *in vitro* without defining the molecular events. In this work we chose to investigate if CHIT1 and YKL40 are involved in their capacity of bone resorption. Since from a preliminary analysis, U266 expressed higher CHIT1 activity and showed higher levels of CHIT1 and YKL40 mRNA than SKM-M1 and MM1 MM cell lines, we chose U266 cells as MM model. By silencing CHIT1 and YKL40 alone with siRNA, we found a reduction of the U266 digestion activity; we also observed an additional effect using the two siRNA in combination. Interestingly, exposure of U266 to BO was able to decrease CHIT1 and YKL40 expression. All these data suggest that BO treatment in MM patients results in an improvement of bone remodeling through the stimulation of bone formation and the suppression of bone resorption not only by OCs but potentially also by PCs; all these BO effects contribute to the antimyeloma efficacy of this drug (Pennisi et al., 2009).

In conclusion, these findings strongly support an evolving concept regarding the role of CHIT1 and YKL40 in MM disease. This is the first study reporting not only that CHIT1 may emerge as an useful serum marker with YKL40 for osteolysis, but also that it is functionally and directly involved in MM osteolytic lesion. Therefore, MM patients with elevated serum levels of CHIT1 and YKL40 may have an increased osteolytic activity and a faster progression of bone disease. BO treatment inhibits osteoclastogenesis by reducing CHIT1 and YKL40 expression. Additional studies to disclose the molecular mechanisms regulating the expression of these chitinases in MM patients and their mechanism of action in the bone degenerative disease would be very important to develop novel strategies of treatment for the management of degenerative skeletal diseases.

### Conflict of Interest Statement

The authors declare that the research was conducted in the absence of any commercial or financial relationships that could be construed as a potential conflict of interest.

## Abbreviations

BO: bortezomib
CHIT1: chitotriosidase
DDs: dentin disks
MM: multiple myeloma
MMP9: Matrix metallopeptidase 9
OCs: osteoclasts
siRNA: small interfering RNA
YKL40: chitinase 3-like-1.
